# Supplementary material for: IDH1 mutation impairs antiviral response and potentiates oncolytic virotherapy in glioma
Source: Nat Commun. 2023 Oct 25;14:6781. doi: 10.1038/s41467-023-42545-3 (PMC10600173; doi:10.1038/s41467-023-42545-3)
Supplement: Supplementary file 1 — Supplementary Information [file 41467_2023_42545_MOESM1_ESM.pdf]

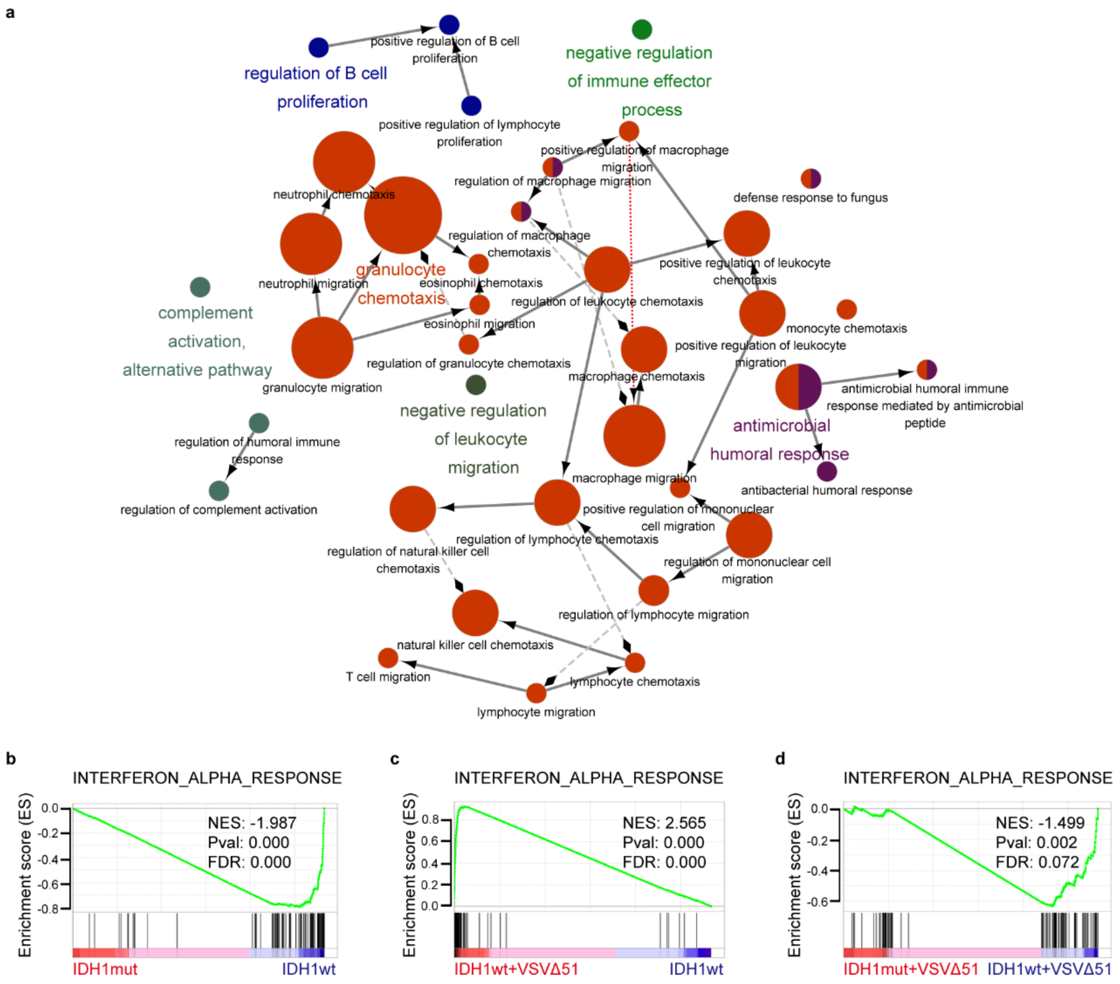

Supplementary Fig. 1 | The innate immune signaling pathways were suppressed in IDH1mut gliomas versus IDH1wt gliomas.

(a) ClueGO analysis showing the interaction networks of enriched biological processes of the downregulated genes in IDH1mut samples versus IDH1wt samples from the glioma patient dataset GSE109857. The size of dots represents the term enrichment significance, and multiple color dots indicate that it involved in multiple biological processes. Functionally related groups partially overlap.

(b-d) GSEA identified the interferon alpha response signature in the three groups of comparisons. NES, normalized enrichment score; Pval, *P* value; FDR, false discovery rate. Pval < 0.05 and FDR < 0.25 were used as cut-off for statistical significance.

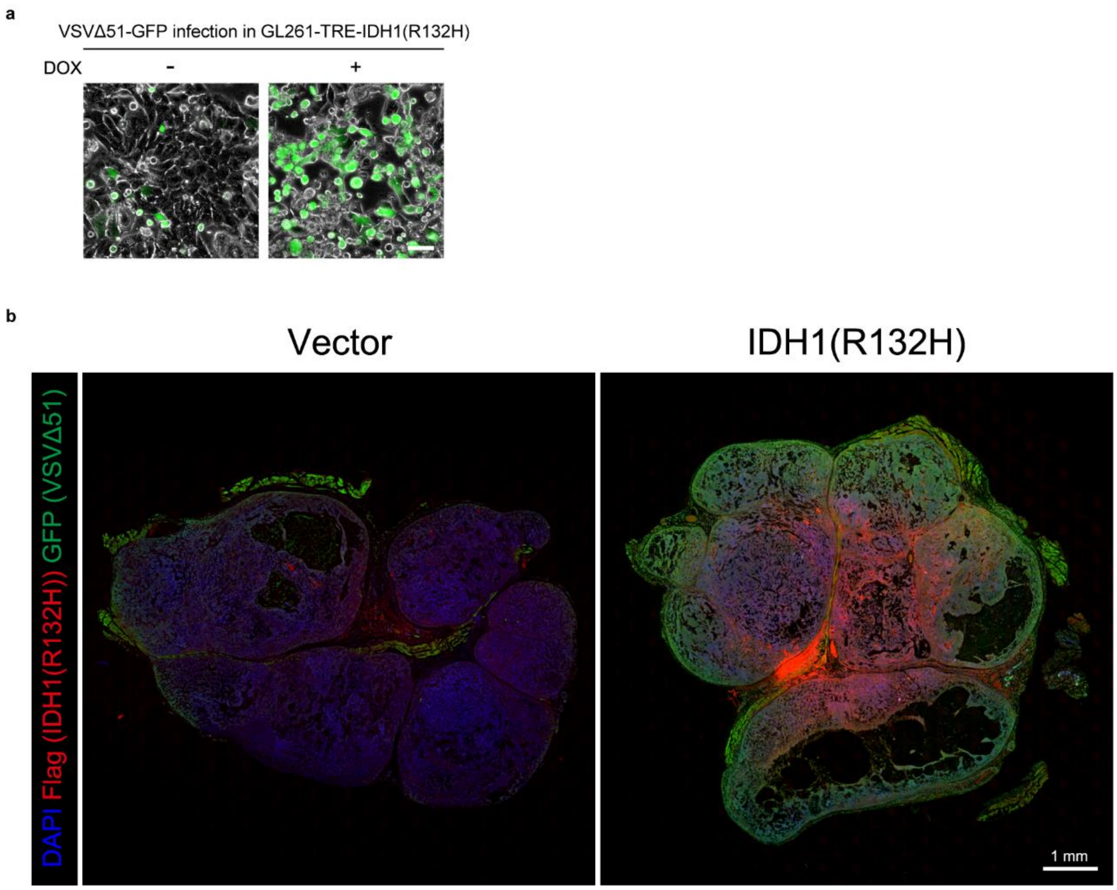

Supplementary Fig. 2 | IDH1 mutation enhances the replication of VSVΔ51 in GL261.

(a) Cells were infected with VSVΔ51–GFP (MOI = 1) infection for 24 hours in the absence or presence of doxycycline (DOX). Phase-contrast and fluorescence microscopy images were captured. Representative images of n = 3. Scale bar, 50 μm.

(b) Whole tumor IF staining showing the expression of GFP (reporter gene for VSVΔ51) and Flag-IDH1(R132H) proteins in tumor tissues from the mice in **Fig. 2j**.

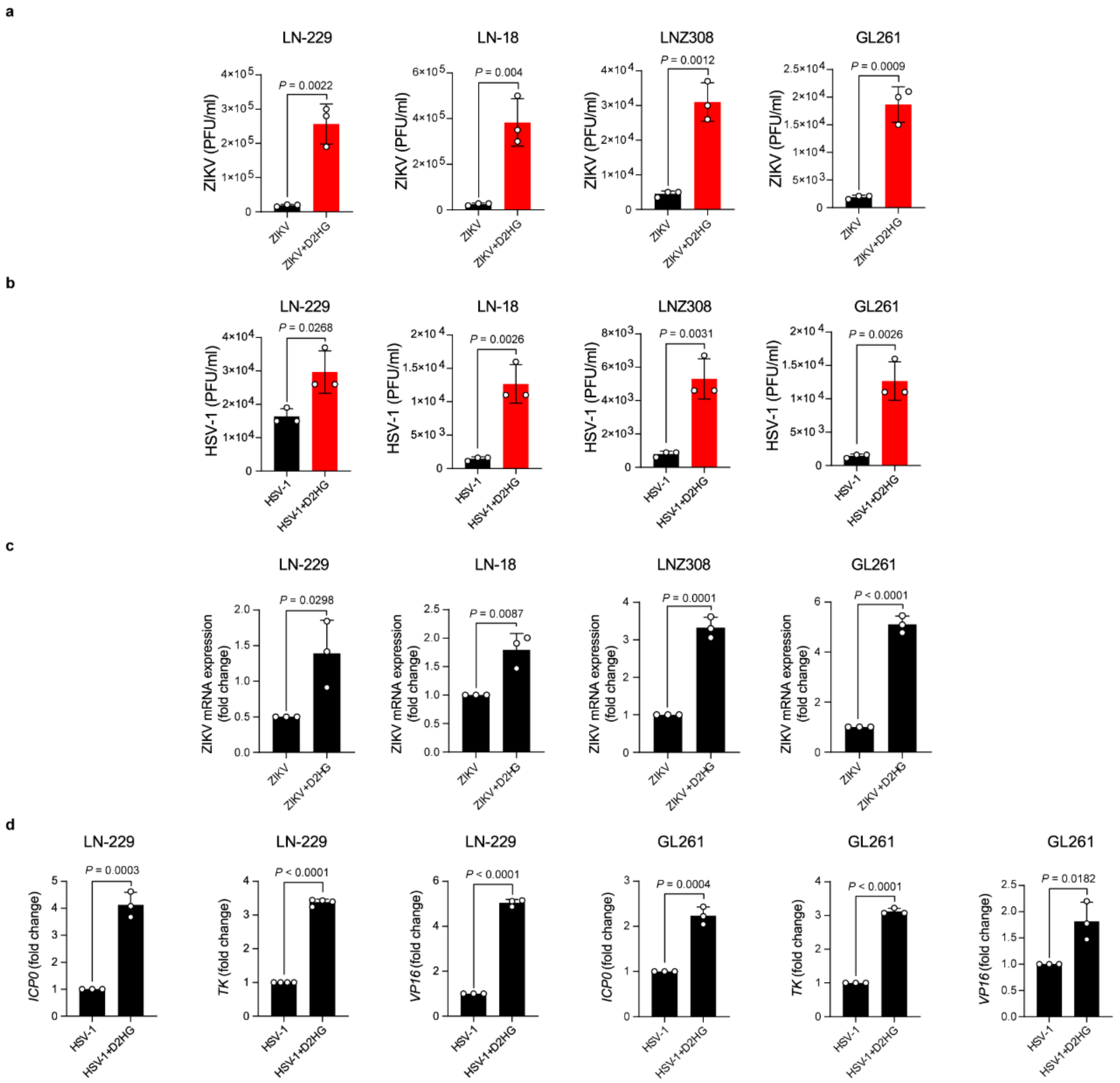

Supplementary Fig. 3 | D2HG promotes the replication of ZIKV and HSV-1 in glioma cells.

(a-d) Cells were pretreated with D2HG (400 µM) for 48 hours, followed by ZIKV (MOI = 0.1) or HSV-1 (MOI = 1) infection. **(a,b)** Corresponding viral titers in supernatants were determined after ZIKV treatment for 48 hours or HSV-1 treatment for 72 hours. **(c,d)** qRT-PCR analysis assessing expression of ZIKV mRNAs or HSV-1 mRNAs encoding ICP0, TK and VP16 after infection for 24 hours. n = 3 or 4 biological replicates.

Data represent the mean ± SD. Statistical significance was determined using two-tailed Student's t-test. Source data are provided in the Source Data file.

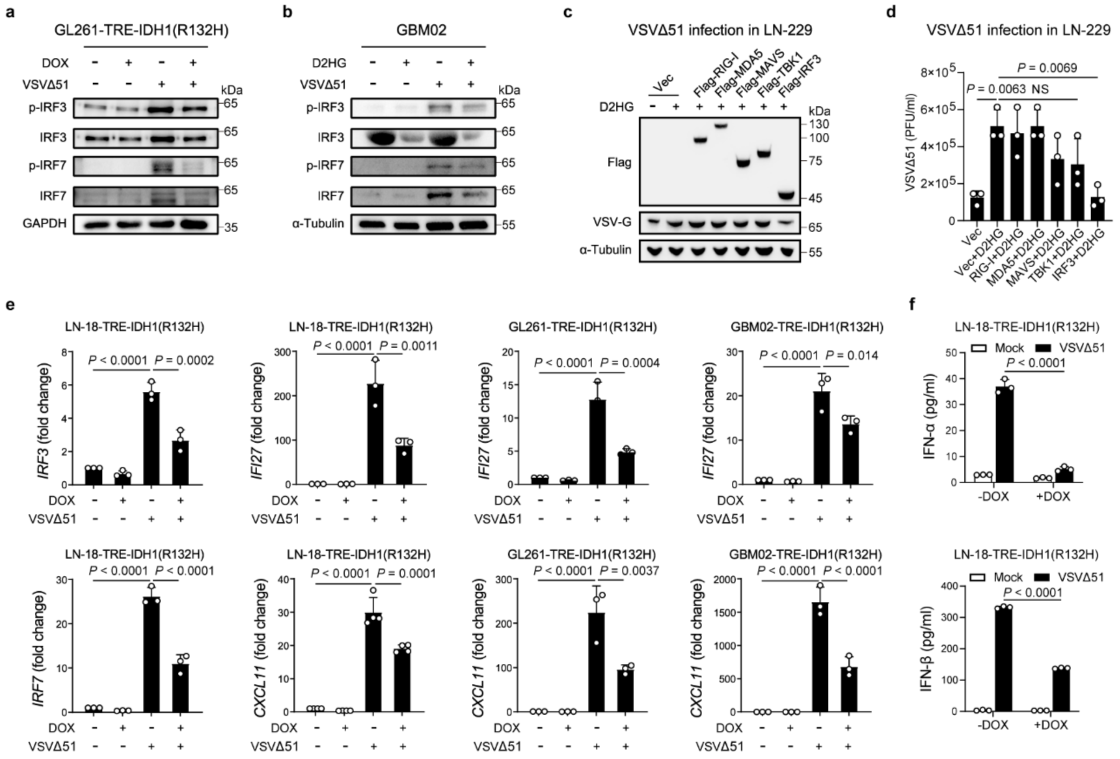

Supplementary Fig. 4 | D2HG enhances VSVΔ51 replication by inhibiting IRF3/7 expression and IFN antiviral responses.

(a,b) Cells pretreated with doxycycline (DOX) **(a)** or D2HG (400 μM) **(b)** for 48 hours and subsequently infected with VSVΔ51 (MOI = 1) for 12 hours. Proteins were examined by western blot.

(c,d) Cells were pretreated with 2HG (400 μM) for 48 hours and then transfected with the indicated plasmids for 24 hours, followed by infection with VSVΔ51 (MOI = 1) for 24 hours. **(c)** Western blot analysis of the indicated proteins. **(d)** Viral titers in supernatants. n = 3 biological replicates. NS, not significant.

(e,f) Cells were pretreated with DOX for 48 hours and subsequently infected with VSVΔ51 (MOI = 1 for LN18, MOI = 10 for GL261, MOI = 0.1 for GBM02) for 12 hours. **(e)** qRT-PCR assessing expression of *IRF3*, *IRF7*, *IFI27* and *CXCL11* mRNA. n = 3 biological replicates. **(f)** Supernatants were collected and assayed by ELISA for IFN-α and IFN-β production. n = 3 biological replicates.

Data represent the mean ± SD. Statistical significance was determined using one-way ANOVA in **(d)**, **(e)**, or two-way ANOVA in **(f)**. Source data are provided in the Source Data file.

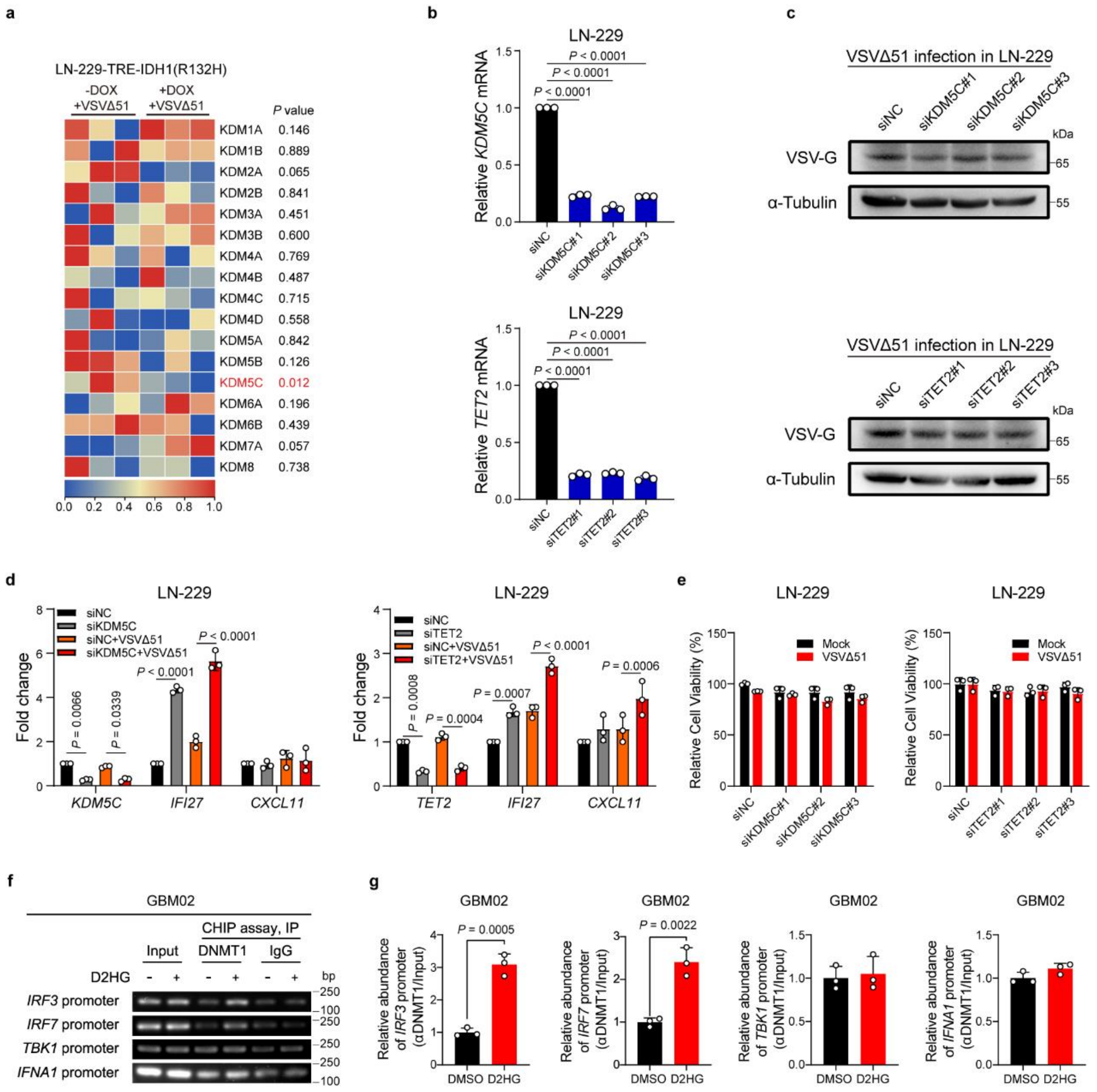

Supplementary Fig. 5 | DNMT1, but not KDM5C or TET2, plays a key role in D2HG-mediated suppression of IFN antiviral responses.

(a) Heatmap for differential expression of transcripts relative to histone lysine demethylases (KDMs). KDM5C was downregulated after doxycycline-induced IDH1 mutation in cells infected with VSVΔ51. n = 3 biological replicates.

(b,c) LN-229 cells were transfected with siRNA targeting KDM5C or TET2 for 48 hours, followed by VSVΔ51 infection (MOI = 1) for 24 hours. **(b)** qRT-PCR analysis of the relative abundance of *KDM5C* or *TET2* transcripts. n = 3 biological replicates. **(c)** Viral protein VSV-G were analyzed by western blot.

(d) LN-229 cells were transfected with siRNA targeting KDM5C or TET2 for 48 hours, followed by VSVΔ51 infection (MOI = 1) for 12 hours. *KDM5C* or *TET2*, *IFI27* and *CXCL11* mRNA levels were analyzed by qRT-PCR. n = 3 biological replicates.

(e) Relative cell viability in the cells after infection for 72 hours. n = 3 biological replicates.

(f) Chromatin immunoprecipitation (ChIP) analysis in GBM02 cells treated with or without D2HG (400 μM, 48 hours).

(g) qRT-PCR analysis of the relative abundance of the indicated promoter segment in the ChIP assays as in **(f)**. n = 3 biological replicates.

Data represent the mean ± SD. Statistical significance was determined using two-tailed Student's t-test in **(a)**, **(d)**, **(g)**, or one-way ANOVA in **(b)**. Source data are provided in the Source Data file.

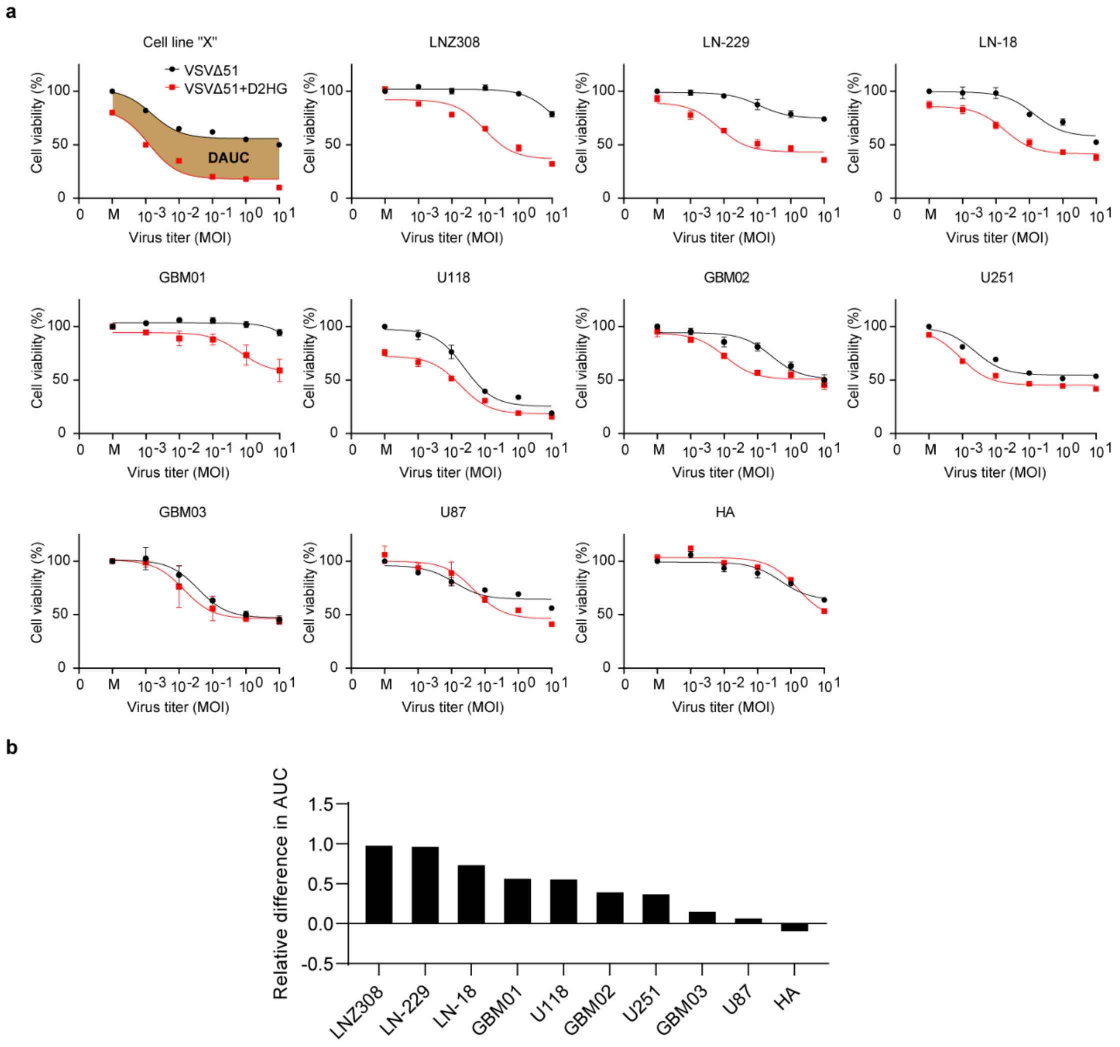

Supplementary Fig. 6 | D2HG improves the oncolytic efficacy of VSVΔ51 in glioma cells.

(a,b) The indicated glioma cell lines (n = 6), patient-derived GBM cell lines (n = 3) and non-cancer cells (normal primary human astrocytes: HA) were pretreated with D2HG (400 µM) for 48 hours, and then infected with increasing titers of VSVΔ51 for 72 hours. **(a)** Dose–response curves were generated for each cell line with or without the pretreatment of D2HG, each point represents the mean ± SD, for n = 3 biological replicates. **(b)** Areas under the curve (AUCs) were calculated using GraphPad Prism software, and then the differences in AUC (DAUC) were calculated according to the formula: $AUC_{VSV\Delta51}$-$AUC_{D2HG+VSV\Delta51}$. The relative difference in AUC were calculated according to the formula: $DAUC_{X}/DAUC_{LNZ308}$.

Source data are provided in the Source Data file.

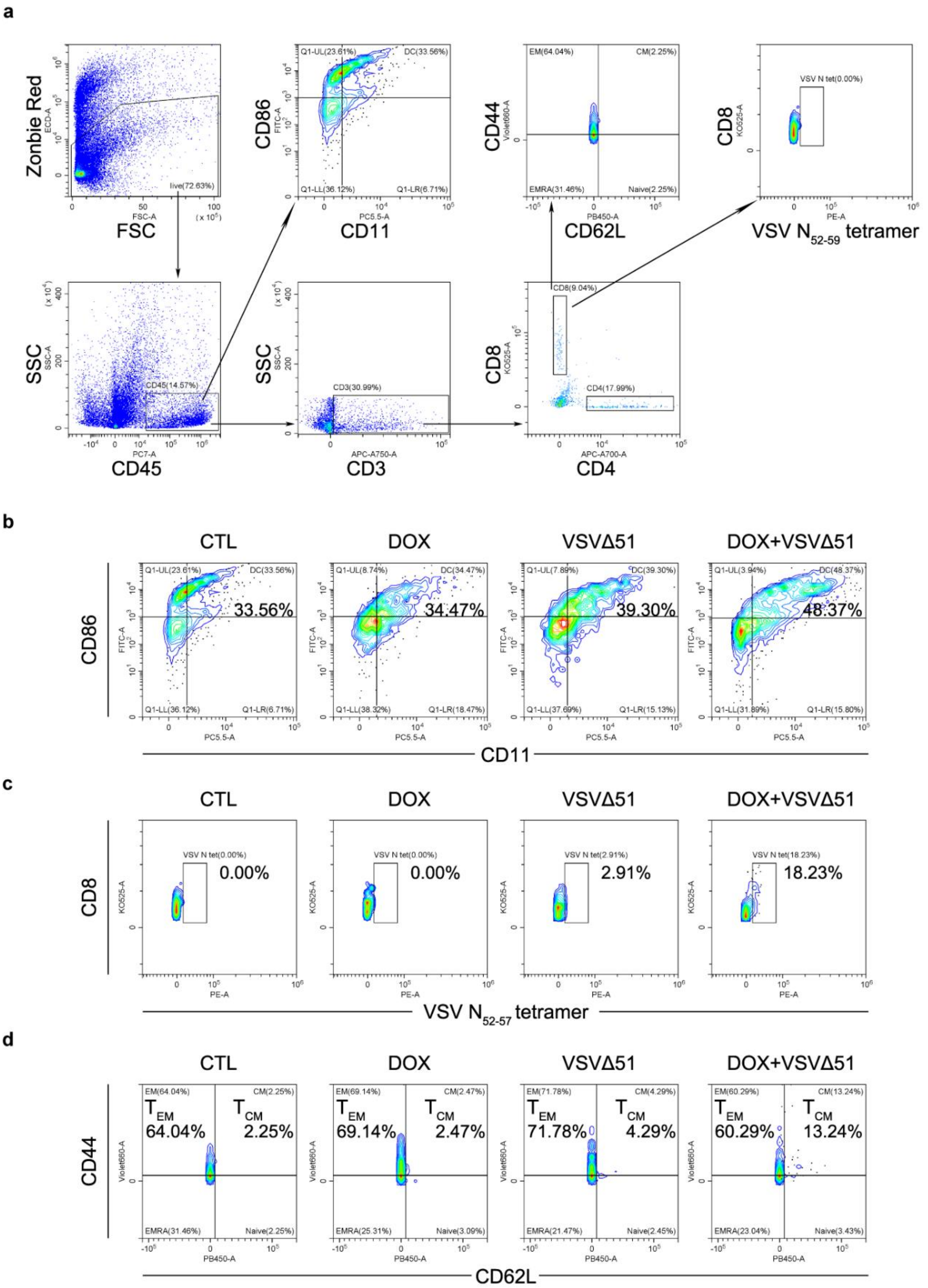

Supplementary Fig. 7 | Gating strategy **(a)** and representative flow cytometry plots **(b-d)** for the assessment of **(b)** $CD11^+$ $CD86^+$ DCs, **(c)** VSVΔ51-specific $CD8^+$ T cells stained with VSV N_{52-57} tetramers, **(d)** effector memory $CD8^+$ T cells ($CD8^+$ T_{EM}, $CD44^+$ $CD62L^-$) and central memory $CD8^+$ T cells ($CD8^+$ T_{CM}, $CD44^+$ $CD62L^+$).

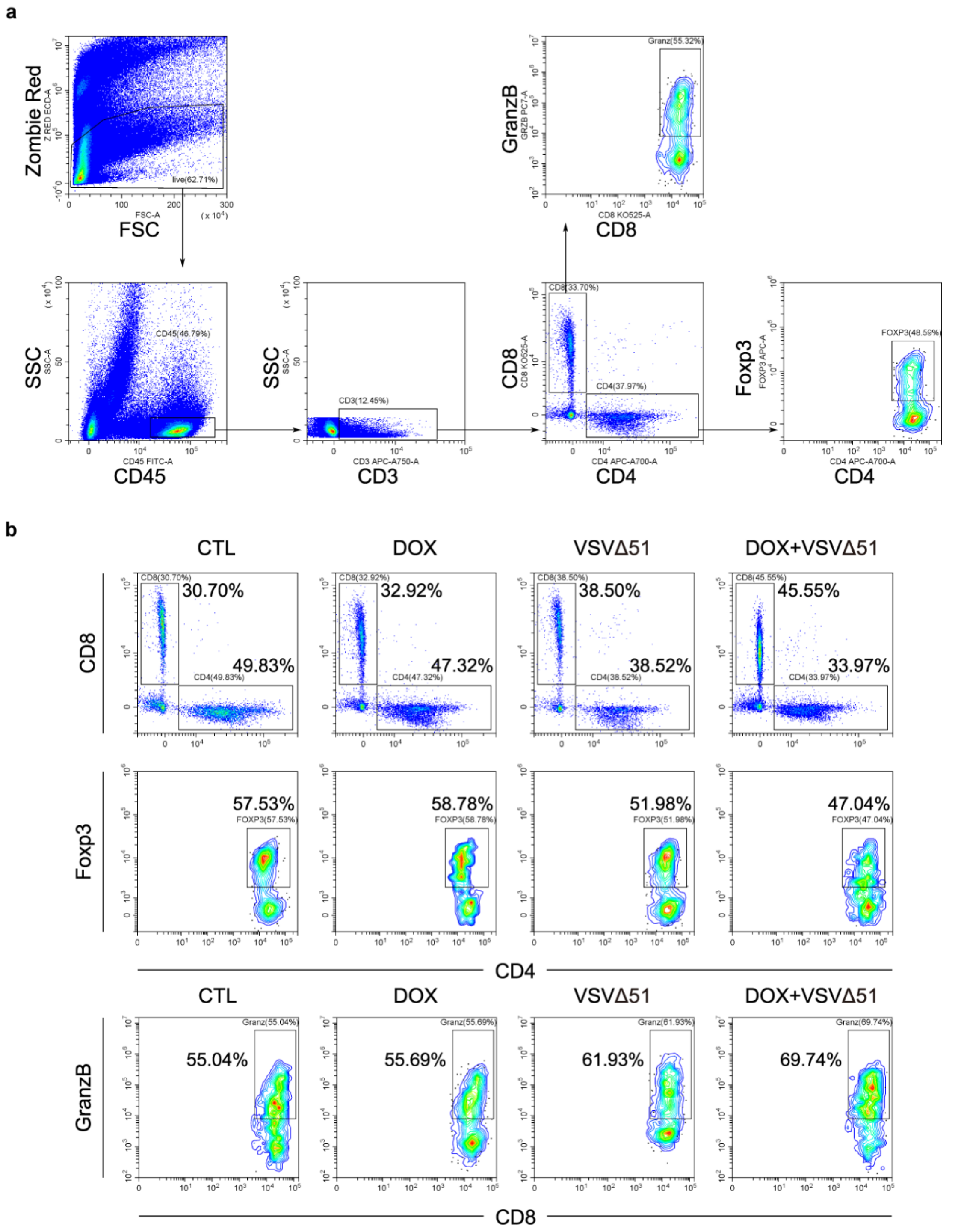

Supplementary Fig. 8 | Gating strategy **(a)** and representative flow cytometry plots **(b)** for the assessment of $CD8^+$ T cells, Tregs ($Foxp3^+$) and the function of $CD8^+$ T cells ($GranzB^+$).

Supplementary Table 1. The sequence of oligonucleotides used in this study.

qPCR primers	F- Forward primer sequences(5'-3')	R- Reverse primer sequence(5'-3')
h. *IRF1*	CTGTGCGAGTGTACCGGATG	ATCCCCACATGACTTCCTCTT
h. *IFI27*	TGCTCTCACCTCATCAGCAGT	CACAACTCCTCCAATCACAACT
h. *CXCL11*	GACGCTGTCTTTGCATAGGC	GGATTTAGGCATCGTTGTCCTTT
h. *BATF2*	AGACCCCAAGGAGCAACA	CAGGGCGAGGTTGTCTTT
h. *UBA7*	TCTCGGGAATTGAGGGAATGG	TAGCCCCACCACGCAAGTA
h. *GBP2*	TTTCCAGCATTTGTGTGGACT	GGGAAGAACTTTCGGATGCAC
h. *TRIM14*	TACATTACAGACGCCATTGGAC	GGGCTGGTTTTCAACAAGGT
h. *CCRL2*	TGGACTGTACTTCGTGGGC	GGACACTTGTAATGATGCCACA
h. *LPAR6*	GGACAATGTACCCAATCACTCTC	ACTTCTCCTGACAGACCAGTTT
h. *OGFR*	TGTGTAGGTATCGGCACAACT	TGTCCTCAATGAAACAGCCGT
h. *IRF3*	AGAGGCTCGTGATGGTCAAG	AGGTCCACAGTATTCTCCAGG
h. *IRF7*	CCCACGCTATACCATCTACCT	GATGTCGTCATAGAGGCTGTTG
h. *KDM5C*	GGGTCCGACGATTTCCTACC	ATGCCCGATTTCTCTGCGATG
h. *TET2*	GATAGAACCAACCATGTTGAGGG	TGGAGCTTTGTAGCCAGAGGT
m. *IRF3*	GAGAGCCGAACGAGGTTCAG	CTTCCAGGTTGACACGTCCG
m. *IRF7*	CCCCAGCCGGTGATCTTTC	CACAGTGACGGTCCTCGAAG
m. *IFI27*	CATCATTGGATTCGGTTCCTGT	CCTTCTTGCTGCTTTGCCTG
m. *CXCL11*	GGCTTCCTTATGTTCAAACAGGG	GCCGTTACTCGGGTAAATTACA
ZIKV. *F10287, R10402*	AGGATCATAGGTGATGAAGAAAAGT	CCTGACAACACTAAGATTGGTGC
HSV. *ICP0*	GGCCCCCTTGTCAACAGA	GGGAGTCGCTGATCACTATGG
HSV. *VP16*	GCGGGGCCGGGATTTACC	CTCGAAGTCGGCCATATCCA
HSV. *TK*	AAGGTCGGCGGGATGAG	CGGCCGCGCGATAC
**siRNA sequences**	**Sequence(5'-3')**	
KDM5C siRNA1	GAGTCAACATCGCCTAAGA	
KDM5C siRNA2	CACGTCCATTTGATAATGA	
KDM5C siRNA3	GCTACAGGCTGAACCTAGA	
TET2 siRNA1	GCAACATAAGCCTCATAAA	

TET2 siRNA2	GTAGCAGTGGAGAGCTACA	
TET2 siRNA3	CCAGTAAACTAGCTGCAAT	
IRF3 siRNA1	CCTCAGATCTGGCTATTGT	
IRF3 siRNA2	GGCTATTGTTTCTGATCCT	
IRF7 siRNA1	CCGAGAACTGGAGGAGTTT	
IRF7 siRNA2	CCAACAGTCTCTACGAAGA	
DNMT1 siRNA1	GGAACTTTGTCTCCTTCAA	
DNMT1 siRNA2	CAATGAGACTGACATCAAA	
**ChIP-qPCR primers**	**F- Forward primer sequences(5'-3')**	**R- Reverse primer sequence(5'-3')**
h. *IRF3* promoter	CTGTGCGAGTGTACCGGATG	ATCCCCACATGACTTCCTCTT
h. *IRF7* promoter	GCTTGTCAGCACTTTCCCTA	GTCGAACTCCCGACCTCA
h. *TBK1* promoter	TGCTCTCACCTCATCAGCAGT	CACAACTCCTCCAATCACAACT
h. *IFNA1* promoter	GACGCTGTCTTTGCATAGGC	GGATTTAGGCATCGTTGTCCTTT
