# Supplementary material for: IDH1 mutation impairs antiviral response and potentiates oncolytic virotherapy in glioma
Source: Nat Commun. 2023 Oct 25;14:6781. doi: 10.1038/s41467-023-42545-3 (PMC10600173; doi:10.1038/s41467-023-42545-3)
Supplement: Supplementary file 3 — Reporting Summary [file 41467_2023_42545_MOESM3_ESM.pdf]

nature portfolio

Corresponding author(s): Haipeng Zhang

Last updated by author(s): Sep 23, 2023

Reporting Summary

Nature Portfolio wishes to improve the reproducibility of the work that we publish. This form provides structure for consistency and transparency in reporting. For further information on Nature Portfolio policies, see our Editorial Policies and the Editorial Policy Checklist.

Statistics

For all statistical analyses, confirm that the following items are present in the figure legend, table legend, main text, or Methods section.

n/a	Confirmed	
☐	☒	The exact sample size (*n*) for each experimental group/condition, given as a discrete number and unit of measurement
☐	☒	A statement on whether measurements were taken from distinct samples or whether the same sample was measured repeatedly
☐	☒	The statistical test(s) used AND whether they are one- or two-sided<br>*Only common tests should be described solely by name; describe more complex techniques in the Methods section.*
☒	☐	A description of all covariates tested
☐	☒	A description of any assumptions or corrections, such as tests of normality and adjustment for multiple comparisons
☐	☒	A full description of the statistical parameters including central tendency (e.g. means) or other basic estimates (e.g. regression coefficient) AND variation (e.g. standard deviation) or associated estimates of uncertainty (e.g. confidence intervals)
☐	☒	For null hypothesis testing, the test statistic (e.g. *F*, *t*, *r*) with confidence intervals, effect sizes, degrees of freedom and *P* value noted<br>*Give P values as exact values whenever suitable.*
☒	☐	For Bayesian analysis, information on the choice of priors and Markov chain Monte Carlo settings
☒	☐	For hierarchical and complex designs, identification of the appropriate level for tests and full reporting of outcomes
☐	☒	Estimates of effect sizes (e.g. Cohen's *d*, Pearson's *r*), indicating how they were calculated

Our web collection on statistics for biologists contains articles on many of the points above.

Software and code

Policy information about availability of computer code

Data collection	No customized software was used for data collection. Real-time PCR data were collected by Bio-Rad CFX96 Real-Time PCR Detection System and associated software; The data of cellular morphology and magnitude of confluence were collected in real time using the IncuCyte Live-Cell Imaging Analysis System and associated software; Western blot data were collected by Tanon 4600 Chemiluminescence Imaging System and associated software; The IHC and H&E sections were digitalized using a Zeiss Axio Scan.Z1 slide scanner and associated software; Microscope data were collected by NIS-Elements viewer 4.20; Cell Viability data and ELISA data were collected by Bio-Rad iMark microplate reader and associated software; Dual-luciferase activity data were collected by the GloMax Luminometer and associated software; Flow cytometric data were collected by CytExpert 2.4. Additional information about software was described in the manuscript or available upon request.
Data analysis	No customized software was used for data analysis. Statistical analyses were performed using Graphpad Prism 8; The clinical data from public databases were analyzed using Limma R-package, GSEA 4.1.0 and ClueGO 2.5.8. The RNA-seq data were analyzed using GSEA 4.1.0 and TBtools; Western blot data and IHC staining data were quantified using Image J 1.51k; Flow cytometric data were analyzed using CytExpert 2.4.

For manuscripts utilizing custom algorithms or software that are central to the research but not yet described in published literature, software must be made available to editors and reviewers. We strongly encourage code deposition in a community repository (e.g. GitHub). See the Nature Portfolio guidelines for submitting code & software for further information.

Data

Policy information about availability of data

All manuscripts must include a data availability statement. This statement should provide the following information, where applicable:

- Accession codes, unique identifiers, or web links for publicly available datasets
- A description of any restrictions on data availability
- For clinical datasets or third party data, please ensure that the statement adheres to our policy

Expression and clinical data of the glioma patient datasets (GSE52942 and GSE109857) was downloaded from NCBI Gene Expression Omnibus (GEO; www.ncbi.nlm.nih.gov/geo). Expression and clinical data of The Cancer Genome Atlas (TCGA) lower grade glioma (LGG) cohort was obtained from UCSC Xena (https://xenabrowser.net/datapages/). The accession number for the raw and processed data of bulk RNA sequencing generated and reported in this paper is GEO: GSE208634. The remaining data generated or analyzed during this study are available within the article, Supplementary Information, or Source Data file. Source data are provided with this paper.

Research involving human participants, their data, or biological material

Policy information about studies with human participants or human data. See also policy information about sex, gender (identity/presentation), and sexual orientation and race, ethnicity and racism.

Reporting on sex and gender	The study did not account for gender.
Reporting on race, ethnicity, or other socially relevant groupings	n/a
Population characteristics	Immunohistochemistry on primary samples was performed on 122 patient samples, and primary GBM cell lines were obtained from 5 patient samples. Ages range from 42 to 65 years.
Recruitment	Tumor samples were collected from patients with newly diagnosed primary brain cancer undergoing surgery at the Sun Yat-sen University Cancer Center. Patients who had received or were receiving other treatments were excluded.
Ethics oversight	This study has received approval from the Institutional Ethics Committee for Clinical Research and Animal Trials of the Sun Yat-sen University Cancer Center (no. B2021-259-01) and written informed consent was obtained from all participants included in the study.

Note that full information on the approval of the study protocol must also be provided in the manuscript.

Field-specific reporting

Please select the one below that is the best fit for your research. If you are not sure, read the appropriate sections before making your selection.

☒ Life sciences ☐ Behavioural & social sciences ☐ Ecological, evolutionary & environmental sciences

For a reference copy of the document with all sections, see nature.com/documents/nr-reporting-summary-flat.pdf

Life sciences study design

All studies must disclose on these points even when the disclosure is negative.

Sample size	For in vitro studies, the experiments were repeated at least 3 times. It is commonly used as the sample size for in vitro studies. For the in vivo experiments, the sample size is determined by the minimum principle of ethical requirements and the tumorigenic capacity of the cancer cells.
Data exclusions	No data were excluded in this study.
Replication	The reproducibility of the data was confirmed in multiple biological replicates as indicated in the figure legends.
Randomization	For in vivo experiments , randomizing were performed such that the average tumor sizes were similar across treatment groups. Except for experiments involving mice, the samples were randomly divided into experimental and control groups.
Blinding	In in vitro experiments utilizing instrumental readouts such as MTT assay, flow cytometry, RNA-sequencing, and western blot, blinding was not implemented due to the inherent objectivity of the detection instruments. However, in cases of potential bias, such as microscopy analysis or in vivo experiments, data collectors were blinded to the experimental groups.

Reporting for specific materials, systems and methods

We require information from authors about some types of materials, experimental systems and methods used in many studies. Here, indicate whether each material, system or method listed is relevant to your study. If you are not sure if a list item applies to your research, read the appropriate section before selecting a response.

Materials & experimental systems

n/a	Involved in the study
☐	☒ Antibodies
☐	☒ Eukaryotic cell lines
☒	☐ Palaeontology and archaeology
☐	☒ Animals and other organisms
☒	☐ Clinical data
☒	☐ Dual use research of concern
☒	☐ Plants

Methods

n/a	Involved in the study
☒	☐ ChIP-seq
☐	☒ Flow cytometry
☒	☐ MRI-based neuroimaging

Antibodies

Antibodies used

Antibodies/Source/Identifier/Dilution ratio
Anti-IDH1(R132H) NewEast 26081 1:1000
Anti-VSV-G Kerafast 8G5F11 1:1000 (IB) 1:100 (IHC)
Anti-Flag Sigma F1804 1:1000 (IB) 1:500 (IF)
Anti-GFP Servicebio GB13227 1:1500
Anti-RIG-I Cell Signaling Technology 3743 1:1000
Anti-MDA5 Cell Signaling Technology 5321 1:1000
Anti-MAVS Cell Signaling Technology 3993 1:1000
Anti-TBK1 Cell Signaling Technology 3504 1:1000
Anti-phosphorylated TBK1 Cell Signaling Technology 5483 1:1000
Anti-IRF3 Abcam ab68481 1:1000 (IB) 1:500 (IHC)
Anti-phosphorylated IRF3 Cell Signaling Technology 29047 1:1000
Anti-IRF7 Cell Signaling Technology 4920 1:1000
Anti-phosphorylated IRF7 Cell Signaling Technology 5184 1:1000
Anti-STAT1 Cell Signaling Technology 14994 1:1000
Anti-phosphorylated STAT1 Cell Signaling Technology 9167 1:1000
Anti-DNMT1 Novus NB100-56519 1:1000 (IB) 1:150 (ChIP)
Anti-Ki-67 Servicebio GB111499 1:200
Anti-cleaved-caspase-3 Servicebio GB11532 1:200
Anti-NKp44 Servicebio GB11615 1:200
Anti-CD45 BioLegend 103108 103114 1:200
Anti-CD3 BioLegend 100330 1:200
Anti-CD4 BioLegend 100536 1:200
Anti-CD8 BioLegend 100752 1:200
Anti-Foxp3 BioLegend 320014 1:200
Anti-Granzyme B BioLegend 372214 1:200
Anti-CD86 BioLegend 105005 1:200
Anti-CD11 BioLegend 117327 1:200
Anti-CD44 BioLegend 103049 1:200
Anti-CD62L BioLegend 104436 1:100
Anti-α-Tubulin Arigo ARG65693 1:5000
Anti-GAPDH Arigo ARG65680 1:5000
H-2Kb VSV NP52–59 RGYVYQGL (PE–labeled tetramer) MBL TS-M529-1 1:50

Validation

The primary antibodies have been validated their respective manufacturers for their respective species, and applications. The validation statement for each primary antibody is available on the manufacturer's website.

Anti-IDH1(R132H) NewEast 26081:
Species: vertebrates; Application: ELISA, WB, IHC, IP; Manufacturer's website: http://www.neweastbio.com.cn/PointMutationAntibody/241

Anti-VSV-G Kerafast 8G5F11:
Species: Tissue, cells or virus corresponding to VSV-G protein from the Indiana serotype; Application: WB, IF, FC; Manufacturer's website: http://kerafast.com.cn/index.php?id=1931&project=product

Anti-Flag Sigma F1804:
Species: all; Application: IHC; Manufacturer's website: https://www.sigmaaldrich.cn/CN/zh/product/sigma/f1804

Anti-GFP Servicebio GB13227:
Species: Human, Mouse, Rat; Application: IHC; Manufacturer's website: https://www.servicebio.cn/search-result?search=GB13227

Anti-RIG-I Cell Signaling Technology 3743:
Species: Human, Mouse, Rat, Hamster, Monkey; Application: WB, IP; Manufacturer's website: https://www.cellsignal.com/products/primary-antibodies/rig-i-d14g6-rabbit-mab/3743

Anti-MDA5 Cell Signaling Technology 5321:
Species: Human, Mouse; Application: WB, IP; Manufacturer's website: https://www.cellsignal.com/products/primary-antibodies/mda-5-d74e4-rabbit-mab/5321

Anti-MAVS Cell Signaling Technology 3993:
Species: Human; Application: WB, IF; Manufacturer's website: https://www.cellsignal.com/products/primary-antibodies/mavs-antibody/3993

Anti-TBK1 Cell Signaling Technology 3504:
Species: Human, Mouse, Rat, Monkey; Application: WB, IP; Manufacturer's website: https://www.cellsignal.com/products/primary-antibodies/tbk1-nak-d1b4-rabbit-mab/3504

Anti-phosphorylated TBK1 Cell Signaling Technology 5483:
Species: Human, Mouse; Application: WB, IP, IF, FC; Manufacturer's website: https://www.cellsignal.com/products/primary-antibodies/phospho-tbk1-nak-ser172-d52c2-xp-rabbit-mab/5483

Anti-IRF3 Abcam ab68481:
Species: Human, Mouse; Application: Flow Cyt (Intra), ICC/IF, WB, IHC-P; Manufacturer's website: https://www.abcam.cn/products/primary-antibodies/irf3-antibody-epr2418y-ab68481.html

Anti-phosphorylated IRF3 Cell Signaling Technology 29047:
Species: Human, Mouse, Rat; Application: WB, IP, IF, FC; Manufacturer's website: https://www.cellsignal.cn/products/primary-antibodies/phospho-irf-3-ser396-d6o1m-rabbit-mab/29047

Anti-IRF7 Cell Signaling Technology 4920:
Species: Human; Application: WB, IP; Manufacturer's website: https://www.cellsignal.cn/products/primary-antibodies/irf-7-antibody/4920?site-search-type=Products&N=4294956287&Ntt=irf7&fromPage=plp

Anti-phosphorylated IRF7 Cell Signaling Technology 5184:
Species: Human; Application: WB; Manufacturer's website: https://www.cellsignal.cn/products/primary-antibodies/phospho-irf-7-ser471-472-antibody/5184?site-search-type=Products&N=4294956287&Ntt=irf7&fromPage=plp

Anti-STAT1 Cell Signaling Technology 14994:
Species: Human, Mouse, Rat, Monkey; Application: WB, IP, IF, FC, IHC, CHIP, C&R; Manufacturer's website: https://www.cellsignal.cn/products/primary-antibodies/stat1-d1k9y-rabbit-mab/14994?site-search-type=Products&N=4294956287&Ntt=14994&fromPage=plp&_requestid=212965

Anti-phosphorylated STAT1 Cell Signaling Technology 9167:
Species: Human, Mouse; Application: WB, IP, IF, FC, IHC, CHIP; Manufacturer's website: https://www.cellsignal.cn/products/primary-antibodies/phospho-stat1-tyr701-58d6-rabbit-mab/9167?site-search-type=Products&N=4294956287&Ntt=9167&fromPage=plp&_requestid=213685

Anti-DNMT1 Novus NB100-56519:
Species: Human, Mouse, Rat, Porcine, Bovine, Sheep; Application: WB, Simple Western, FC, ICC/IF, IHC, IP, ChIP, CyTOF-ready, IHC, KD; Manufacturer's website: https://www.novusbio.com/products/dnmt1-antibody-60b12201_nb100-56519

Anti-Ki-67 Servicebio GB111499:
Species: Human, Mouse, Rat; Application: IHC/IF, ICC/IF; Manufacturer's website: https://www.servicebio.cn/goodsdetail?id=3931

Anti-cleaved-caspase-3 Servicebio GB11532:
Species: Human, Mouse, Rat; Application: IHC/IF; Manufacturer's website: https://www.servicebio.cn/goodsdetail?id=1271

Anti-NKp44 Servicebio GB11615:
Species: Human, Mouse; Application: IHC, IF; Manufacturer's website: https://www.servicebio.cn/goodsdetail?id=709

Anti-CD45 BioLegend 103108:
Species: Mouse; Application: FC; Manufacturer's website: https://www.biolegend.com/en-us/products/fitc-anti-mouse-cd45-antibody-99

Anti-CD45 BioLegend 103114:
Species: Mouse; Application: FC; Manufacturer's website: https://www.biolegend.com/en-us/products/pe-cyanine7-anti-mouse-cd45-antibody-1903

Anti-CD3 BioLegend 100330:
Species: Mouse; Application: FC; Manufacturer's website: https://www.biolegend.com/en-us/products/apc-cyanine7-anti-mouse-

cd3epsilon-antibody-6070

Anti-CD4 BioLegend 100536:
Species: Mouse; Application: FC; Manufacturer's website: https://www.biolegend.com/en-us/products/alexa-fluor-700-anti-mouse-cd4-antibody-3386

Anti-CD8 BioLegend 100752:
Species: Mouse; Application: FC; Manufacturer's website: https://www.biolegend.com/en-us/products/brilliant-violet-510-anti-mouse-cd8a-antibody-7992

Anti-Foxp3 BioLegend 320014:
Species: Mouse; Application: FC; Manufacturer's website: https://www.biolegend.com/en-us/products/alexa-fluor-647-anti-mouse-rat-human-foxp3-antibody-2892

Anti-Granzyme B BioLegend 372214:
Species: Human, Mouse; Application: FC; Manufacturer's website: https://www.biolegend.com/en-us/products/pe-cyanine7-anti-humanmouse-granzyme-b-recombinant-antibody-15582

Anti-CD86 BioLegend 105005:
Species: Mouse; Application: FC; Manufacturer's website: https://www.biolegend.com/en-us/products/fitc-anti-mouse-cd86-antibody-254

Anti-CD11 BioLegend 117327:
Species: Mouse; Application: FC; Manufacturer's website: https://www.biolegend.com/en-us/products/percp-cyanine5-5-anti-mouse-cd11c-antibody-4258

Anti-CD44 BioLegend 103049:
Species: Human, Mouse; Application: FC; Manufacturer's website: https://www.biolegend.com/en-us/products/brilliant-violet-650-anti-mouse-human-cd44-antibody-8923

Anti-CD62L BioLegend 104436:
Species: Mouse; Application: FC; Manufacturer's website: https://www.biolegend.com/en-us/products/brilliant-violet-421-anti-mouse-cd62l-antibody-7164

Anti-α-Tubulin Arigo ARG65693:
Species: Human, Mouse, Rat; Application: FC, ICC/IF, IHC-P, WB; Manufacturer's website: https://www.arigobio.cn/anti-alpha-Tubulin-antibody-ARG65693.html

Anti-GAPDH Arigo ARG65680:
Species: Human, Mouse, Rat; Application: ICC/IF, IHC-P, WB; Manufacturer's website: https://www.arigobio.cn/anti-GAPDH-antibody-ARG65680.html

H-2Kb VSV NP52–59 RGYVYQGL (PE–labeled tetramer) MBL TS-M529-1:
Species: Mouse; Application: FC; Manufacturer's website: http://www.mbl-chinawide.cn/search012?keyword=RGYVYQGL

Eukaryotic cell lines

Policy information about cell lines and Sex and Gender in Research

Cell line source(s)	GL261, U251, and U87 cells were generously gifted by Prof. Guangmei Yan (Zhongshan School of Medicine, Sun Yat-sen University). LN-18, LN-229, U118, and LNZ308 cells were generously gifted by Prof. Qi Qi (School of Medicine, Jinan University). Primary patient-derived GBM cells were generously gifted by Dr. Hao Duan (Sun Yat-sen University Cancer Center). GBM01 and GBM04 cell lines are derived from male patients. GBM02, GBM03 and GBM05 cell lines are derived from female patients.
Authentication	All cell lines were authenticated by STR profiling.
Mycoplasma contamination	All cell lines used in experiments were tested negative for mycoplasma contamination.
Commonly misidentified lines (See ICLAC register)	No commonly misidentified cell lines were used.

Animals and other research organisms

Policy information about studies involving animals; ARRIVE guidelines recommended for reporting animal research, and Sex and Gender in Research

Laboratory animals	Four- to six-week-old female BALB/c-nu/nu mice and four-week-old female C57BL/6 mice were purchased from GemPharmatech Co. (Jiangsu, China). Animals were housed in SPF facilities in the experimental animal center of Jinan University. Mice were housed at an ambient temperature of 22-24°C, humidity-controlled environment at 40%-70% under a 12-hour light/dark cycle with ad libitum

	access to water and food. Animal care and handling procedures were in accordance with the Institutional Animal Care and Use Committee (IACUC) protocol and were approved by the Jinan University Institutional Review Board.
Wild animals	No wild animals were used in this study.
Reporting on sex	Female mice in different litters are usually less likely to fight and easier to be raised in groups, which is conducive to the growth of tumors and random grouping.
Field-collected samples	No field-collected samples were used.
Ethics oversight	The mouse experiments were performed under the protocol approved by the Laboratory Animal Ethics Committee of Jinan University (IACUC no. 20210816-04; 20230205-07; 20230205-12).

Note that full information on the approval of the study protocol must also be provided in the manuscript.

Flow Cytometry

Plots

Confirm that:

☒ The axis labels state the marker and fluorochrome used (e.g. CD4-FITC).

☒ The axis scales are clearly visible. Include numbers along axes only for bottom left plot of group (a 'group' is an analysis of identical markers).

☒ All plots are contour plots with outliers or pseudocolor plots.

☒ A numerical value for number of cells or percentage (with statistics) is provided.

Methodology

Sample preparation	Brain tumor quadrants were harvested, minced, incubated with a Brain Tumor Dissociation Kit (130-095-942, Miltenyi), triturated, passed through a 70 mm screen, resuspended in FACS buffer (2% inactivated fetal calf serum in PBS), and stained with fluorochrome-conjugated anti-mouse antibodies from BioLegend. A Zombie Red Fixable viability kit (BioLegend) was used to stain dead cells. We followed a 'no-wash' sequential staining protocol (BioLegend) to stain dead cells and for surface staining. Intracellular Foxp3 staining was performed following the Foxp3 intracellular staining protocol (BioLegend). For single-color compensation controls, compensation particles (552845, BD) were used and stained with each fluorescently conjugated antibodies according to the manufacturer's instructions. For the Zombie Red assay, cells from the nontumor and tumor quadrants, respectively, were used as single-color compensation controls.
Instrument	Cytoflex flow cytometer (Beckman Coulter)
Software	CytExpert 2.4
Cell population abundance	There's no sorting or purification.
Gating strategy	Schematic Gating Strategies are provided in Supplementary Fig. 7 and 8.

☒ Tick this box to confirm that a figure exemplifying the gating strategy is provided in the Supplementary Information.
